# Feline Leukemia Virus p27 Antigen Concentration and Proviral DNA Load Are Associated with Survival in Naturally Infected Cats

**DOI:** 10.3390/v13020302

**Published:** 2021-02-15

**Authors:** Melissa J. Beall, Jesse Buch, Genevieve Clark, Marko Estrada, Andrei Rakitin, Natascha T. Hamman, Monica K. Frenden, Ellen P. Jefferson, E. Susan Amirian, Julie K. Levy

**Affiliations:** 1 IDEXX Laboratories, Inc., Westbrook, ME 04092, USA; jesse-buch@idexx.com (J.B.); genevieve-clark@idexx.com (G.C.); marko-estrada@idexx.com (M.E.); andrei-rakitin@idexx.com (A.R.); 2 Austin Pets Alive!, Austin, TX 78703, USA; nth4@utexas.edu (N.T.H.); monica.frenden@americanpetsalive.org (M.K.F.); ellen.jefferson@austinpetsalive.org (E.P.J.); ea25@rice.edu (E.S.A.); 3 Maddie’s Shelter Medicine Program, University of Florida, Gainesville, FL 32608, USA; levyjk@ufl.edu

**Keywords:** FeLV, progressive, regressive, survival, diagnostics, quantitative, longitudinal

## Abstract

Longitudinal studies of cats naturally infected with feline leukemia virus (FeLV) are important for understanding disease outcomes. Levels of p27 antigen and copy numbers of proviral DNA have been associated with FeLV-infection courses. The purpose of this prospective study was to establish cutoff values for p27 antigen concentration and proviral DNA load that distinguished high positive from low positive groups of cats and to evaluate an association with survival. At enrollment, 254 cats were tested by point-of-care and microtiter plate enzyme-linked immunosorbent assays (ELISAs) for p27 antigen and real-time polymerase chain reaction (PCR) for proviral DNA. The 127 positive cats were retested monthly for six months and monitored for survival over the four-year study. A receiver operating characteristic-based analysis of samples with concordant or discordant qualitative results for p27 antigen and proviral DNA was used to establish cutoff values, and when applied to test results at enrollment for classifying cats as high positive or low positive, a significant difference in survival was observed. High positive cats had a median survival of 1.37 years (95% CI 0.83–2.02) from time of enrollment, while most low positive cats were still alive (93.1% survival). Quantitative results for p27 antigen concentration and proviral DNA load were highly correlated with survival times in FeLV-infected cats.

## 1. Introduction

Retroviruses continue to be important pathogens of domestic cats, and studies that further the understanding of disease outcomes are valuable for informing decisions in patients with these infections. Retroviruses are unique in that they contain the necessary enzymes to make a DNA copy of their RNA genome and integrate those genes into the chromosomes of an infected cell at the time of cell division. Cells arising from that infected cell inherit the viral genes, thereby maintaining the infection. Feline leukemia virus (FeLV), a gammaretrovirus, is one of four major types of retroviruses that are known to infect domestic cats. From the time of its discovery in 1964, FeLV has been known to predispose infected cats to the development of life-threatening diseases related to bone marrow dyscrasia (e.g., anemia and other cytopenias), neoplastic transformation (e.g., lymphoma and leukemia), immunoproliferation (e.g., uveitis and polyarthritis), or immunosuppression (e.g., opportunistic infections) resulting in a reduced lifespan [1,2,3,4]. In recent years, however, both serosurveys and individual case reports have suggested that some cats with FeLV infections survive longer than the three to five years previously described, perhaps in part due to an effective immune response that limits the extent of the infection [5,6,7].

When evaluated at any given point in time, cats infected with FeLV may be classified into one of four clinical courses, i.e., abortive, focal, regressive, or progressive [1,4,8]. Abortive infections are eliminated by an effective immune response prior to the virus successfully infecting actively dividing cells, and this prevents the integration of viral genes into the host chromosomes. Under natural circumstances, cats with abortive infections are difficult to distinguish from uninfected cats because both groups produce negative results on routine screening tests for viral antigens [9]. If the virus is successful at infecting lymphocytes and monocytes in regional lymph nodes at the point of exposure, including actively dividing cells, these infected cells may, then, spread the virus to other lymphoid organs including the spleen, lymph nodes, and gut-associated lymphoid tissues, as well as the bone marrow during the primary viremia. Focal infections, considered to be rare, result when the virus becomes sequestered in a particular tissue and low-level viral replication or transcription continues within infected cells such that p27 antigen may still be detected in peripheral circulation [10,11,12,13,14,15,16]. Regressive infections occur when the immune system controls the infection either before or shortly after infection of the bone marrow, thereby limiting the spread of the virus within the body and minimizing a secondary viremia [4,8,17]. In the absence of effective immune control, a secondary viremia occurs following infection of bone marrow progenitor cells. This allows for infection of the epithelial tissues responsible for viral shedding. The resultant persistent viremia is characteristic of a progressive infection. For any cat that becomes infected, the effectiveness of the ongoing immune response to the virus may influence the clinical course of the infection over time and alter clinical outcomes [5,10,18]. In general, focal and regressive FeLV infections are associated with minimal viral shedding and a lower risk of developing FeLV-associated disease, whereas progressive infections are more likely to result in clinical disease and early death [1,4,8,19]. 

The different clinical courses of an FeLV infection have been shown to be associated with levels of FeLV-specific nucleic acids and proteins that can be measured in samples from an infected cat. For instance, the integration of a DNA copy of the FeLV viral genome into an infected host-cell chromosome allows for the detection of proviral DNA by quantitative, real-time polymerase chain reaction (PCR). These PCR assays have been designed to target the U3 region of the 5′ long terminal repeat (LTR) making them specific for exogenous, or the infectious, FeLV, and allowing detection of the infection even in the absence of viral replication or viremia [20,21]. The core protein of FeLV, p27, is a soluble antigen that is produced in great excess which makes it ideal for detection by immunoassays. Several of the immunoassays that detect p27 antigen in cats employ a pair of high affinity, monoclonal antibodies that, when optimized in an enzyme-linked immunosorbent assay (ELISA), can be very sensitive and specific [22,23,24]. Studies in both experimentally infected and naturally infected cats have demonstrated a strong association between the copy numbers of proviral DNA and p27 antigen level in the circulation and clinical course of infection [5,25]. These studies have demonstrated that cats with progressive infections tend to have higher proviral loads and levels of p27 antigen, while cats with regressive infections tend to have lower proviral loads and levels of p27 antigen. These differences likely contribute to the observation from earlier studies that cats with progressive infections tend to have concordant results between p27 antigen and proviral DNA (both positive), while cats with regressive or focal infections are often recognized as having discordant test results (one positive and one negative) [14,26]. When the concentration of p27 antigen is low or there are a few copy numbers of proviral DNA, either of these measures could fall below the level of detection in the respective assay leading to discordant results with the potential for variation over time. 

Given that focal and regressive infections are associated with lower amounts of p27 antigen and copy numbers of proviral DNA as compared with progressive infections, discordant results between these two methods may be a consequence of the biology of the disease, and also the ability of these assays to consistently produce a positive qualitative result near their limit of detection. When fully quantitative assays for p27 antigen and proviral DNA were applied to a convenience population of commercial diagnostic laboratory samples, a positive correlation between the concentration of p27 antigen and the copy numbers of proviral DNA was observed, and discordant results were more numerous at the lower end of the quantitative range [27]. The purpose of this study was to establish cutoff values for the concentration of p27 antigen and the copy numbers of proviral DNA that could distinguish high positive versus low positive cats and determine if the classification of cats by these cutoff values would be associated with survival during the four-year study period.

## 2. Materials and Methods

### 2.1. Population/Study Design 

The study was conducted in collaboration with a large shelter offering an FeLV adoption program (Austin Pets Alive!, Austin, TX, USA) [28] and approved by the Institutional Animal Care and Use Committee (IACUC) aof the University of Florida, Maddie’s Shelter Medicine Program (protocol 201909584, 10 July 2016). Cats were individually screened at the time of shelter admission for the presence of p27 antigen using EDTA-anticoagulated whole blood with a point-of-care (POC) ELISA test (SNAP^®^ FIV/FeLV Combo Test, IDEXX Laboratories, Inc., Westbrook, ME, USA). Screening for FeLV p27 antigen is a recommended protocol even though some antigen-negative cats with FeLV infections could be missed due to recent exposure to the virus or having established regressive infections [17,29,30]. Cats that tested positive for p27 antigen on the intake test were enrolled consecutively in the FeLV-positive group if they were estimated to be at least 8 weeks of age, weighed at least 2 pounds, and were socialized enough for humane handling. If the time between the initial intake test and enrollment in the study was >14 days, the screening test was repeated to confirm ongoing eligibility for the study. A control population of p27 antigen negative age- and sex-matched unrelated cats was enrolled in parallel using 1:1 matching to a cat in the FeLV-positive group. Cats were removed from the study if, based upon their temperament, they were deemed too stressed for regular examination and blood collection (4/131 excluded). 

Cats assigned to the FeLV-positive group based on p27 antigen screening at the time of admission had blood samples collected again at the time of enrollment (time 0) and monthly for the following six months (time points 1–6). Study cats were placed in foster homes with instructions to return monthly for sample collection and were formally adopted at the end of this phase of the study. Because it was not feasible to monitor the adopted, negative control cats over six months, cats assigned to the negative control group based on their initial p27 antigen screening result only had a single blood sample collected at the time of enrollment (time 0). At each time point, an EDTA-anticoagulated whole blood sample (3 mL) was obtained and stored refrigerated until it was sent on ice packs to IDEXX (IDEXX R&D, Westbrook, ME). Within seven days of blood collection, an aliquot of the whole blood (0.5 mL) was used for both PCR testing and the POC ELISA test at the laboratory. The remainder of the sample was centrifuged for plasma recovery and stored frozen at −20 °C, for batch processing by the microtiter plate ELISA. Laboratory personnel performing the testing were blinded to all patient information.

Enrollment occurred during the first 12 months of the four-year study. Cats assigned to the FeLV-positive group were followed for 3–4 years depending upon their date of enrollment. After completion of the initial six months of the study, pet owners were contacted by a member of the shelter or university at approximately three-month intervals to inquire on the status of their cat(s). If the cat was still alive, the contact date was recorded. If the cat was deceased, the date of death was recorded. Outcome interval was calculated from the date of enrollment to the date of death or most recent contact if still alive.

### 2.2. Enzyme-Linked Immunosorbent Assay (ELISA) Testing

The POC ELISA was performed, according to manufacturer’s instructions, using EDTA-anticoagulated whole blood samples. The microtiter plate ELISA that was used in the current study is a commercially available reference laboratory assay (FeLV Antigen by ELISA, IDEXX Laboratories, Inc., Westbrook, ME, USA) that was performed using the reserved plasma samples. Both test platforms use the same pair of monoclonal antibodies for detecting FeLV p27 antigen, and the performance of each assay has been described previously [23,31]. The microtiter plate ELISA includes orthogonal screening and confirmatory protocols for high specificity. Samples with an optical absorbance (A650) above a predetermined cutoff calculated by the ratio of sample to positive control (S/P) signal were considered to be positive and were reflexed to the confirmatory assay. The confirmatory assay evaluates the specificity of the antibody binding that is detected in the screening assay by using antisera raised against the virus. The confirmatory assay is performed in the same way as the screening assay however, the patient’s sample is preincubated with antisera raised against FeLV that will complex FeLV-related proteins and prevent them from binding in the ELISA. When compared to the patient’s sample diluted with an unrelated antiserum, a 50% or greater reduction in absorbance between the two wells of the assay is considered to be specific binding and confirms the presence of FeLV p27 antigen. In the current study, this same microtiter plate ELISA was performed with quantitation, as previously described, using a panel of recombinant p27 calibrators to determine the concentration of p27 antigen in the sample [27]. The resulting limit of quantitation was estimated to be 1 ng/mL, while the limit of detection for the assay was estimated to be 0.2 ng/mL. This limit of quantitation allowed the confirmatory assay to have sufficient signal to confidently evaluate a 50% or greater reduction in absorbance between the two wells. 

### 2.3. Real-Time Polymerase Chain Reaction (PCR)

Total nucleic acid was extracted from each anticoagulated whole blood sample and tested by real-time PCR (FeLV Quant RealPCR^TM^ Test, IDEXX Laboratories, Inc.) for the presence of FeLV proviral DNA using primers and probes specific for the exogenous form of FeLV [20]. Automated nucleic acid extractions were performed using guanidine thiocyanate-based lysis solution with 90 μL of whole blood. From an eluate of 150 μL, a 5 μL aliquot was added to a 12.6 μL PCR reaction. The quantitative real-time PCR assay includes six internal quality controls and utilizes standardized instrumentation that allows an optimized batch of master mix to be calibrated to a standard curve which is generated from serial dilutions of a synthetic oligonucleotide positive control [27]. The dynamic range of the assay covers 7 orders of magnitude and can detect one copy of the standard per reaction which equates to a limit of quantitation of 300 proviral DNA equivalents/mL of whole blood [27].

### 2.4. Statistical Analysis

A receiver operating characteristic (ROC) analysis, adjusted to account for correlation between repeated measurements (observations) from the same cat performed over the course of study, was used to establish the point estimates and 95% confidence intervals for the p27 antigen concentration and the FeLV proviral DNA loads that provided the optimal discrimination (based on Youden criteria) between samples with qualitative concordant (both positive) and qualitative discordant (one positive and one negative) results [32,33]. All samples with positive qualitative results for p27 antigen by microtiter plate ELISA or proviral DNA by real-time PCR were included in the analysis (*n* = 694). Samples were classified as concordant when the p27 antigen and proviral DNA results were both positive. Discordant samples were classified as either p27 antigen positive/PCR negative or p27 antigen negative/PCR positive. To meet the requirement of independence, two non-overlapping sets of 250 observations were randomly selected (using simple random sampling) from the concordant data. One concordant sample set was combined with the p27 antigen positive/PCR negative discordant sample set, and the second concordant sample set was combined with the p27 antigen negative/PCR positive discordant sample set. Within each combined sample set, two smaller subsets of equal size and independent observations suitable for ROC analysis were generated (see Supplemental Data for full description). ROC analysis was performed based on Firth (penalized likelihood) logistic regression to reduce small sample bias, and to get finite and consistent estimates, even in the case of full data separation [34]. Youden cutoffs for p27 antigen concentration and proviral DNA loads were iteratively determined for the respective sample subsets. Using the qualitative concordant/discordant sample classification, a first iteration p27 antigen concentration ROC analysis was performed on the first subset of ”concordant positive and p27 antigen positive/PCR negative discordant” data, Youden cutoffs calculated, and samples in the second subset of ”concordant positive and p27 antigen positive/PCR negative discordant” data reclassified based on the calculated cutoff. Then, these newly classified samples were used in the second iteration ROC analysis on the second subset to produce a new cutoff that was used to reclassify the samples in the first subset. This procedure continued until convergence criterion (≤0.001) for relative difference (defined as absolute difference divided by mean) in consecutively calculated cutoffs was satisfied. The choice of convergence criteria was based on the expected precision of p27 antigen concentration of ±0.1 ng/mL and log proviral DNA copies/mL of ±0.1. If the ”odd” (first, third, etc.) iteration cutoff resulted in a full data separation (ROC AUC = 1) of the second subset, or if the ”even” (second, fourth, etc.) iteration cutoff resulted in a full data separation of the first subset, the iteration process stopped, and that cutoff was chosen. 

A similar analysis was performed for the ”concordant positives and p27 antigen negative/PCR positive discordant” sample set using the previously calculated p27 antigen cutoff value and proviral DNA logs of copies/mL to improve normality of distribution. Thus, each random partition of the concordant data was used to produce a single pair of p27 antigen and proviral DNA cutoffs. The described analysis was repeated 500 times, each with a new 250 + 250 pair of concordant sample sets, to produce a distribution of 500 pairs of p27 antigen and proviral DNA cutoff values. The distributions of calculated cutoffs were used to determine the point estimates (distribution median) and 95% confidence intervals of the cutoff values for the p27 antigen concentration and proviral DNA load.

The cutoff values for p27 antigen concentration and FeLV proviral DNA load obtained from the ROC analysis were applied to all time 0 samples in the FeLV-positive group (*n* = 127) and were evaluated for an association with survival. Cats were classified as high positive if both the quantitative p27 antigen concentration and proviral DNA loads were greater than their respective cutoff values. Cats were classified as low positive if one or both qualitative results were positive, but the quantitative values were equal to or below the cutoffs determined by the ROC analysis. If a cat had concordant qualitative positive results for p27 antigen and proviral DNA but one result was above and one result was below the determined cutoff values from the ROC analysis, then the cat was classified according to the proviral DNA load (five cats in this group). Cats could also be classified as presumed-negative if both the p27 antigen microtiter plate ELISA and proviral DNA PCR results were negative (indicating that the analyte was either absent or below the limits of quantitation) at time 0 (eight cats in this group). This combination of negative results for a cat that has previously tested positive for p27 antigen with an POC test could be interpreted as a false positive screening result in an uninfected cat or could indicate FeLV exposure resulting in a focal or regressive infection. A Kaplan–Meier analysis with a log-rank test used to assess differences in survival curves characteristic of high positive, low positive, and presumed-negative classified cats was performed. Statistical significance was assessed at *p* < 0.05. All statistical analyses were performed using SAS/STAT software, Version 9.4 (©SAS Institute Inc., Cary, NC, USA).

## 3. Results

### 3.1. Population

During the study enrollment period, 5834 cats were admitted to the shelter (3146 were estimated to be 8 weeks of age and older), and 196 had positive p27 antigen results using anticoagulated whole blood on the POC ELISA. A total of 131 cats with positive POC p27 antigen test results for FeLV and 131 age- and sex-matched control cats with negative POC p27 antigen test results for FeLV on tests performed in the shelter were included in the study. Each cat was assigned to the FeLV-positive or FeLV-negative control group based on the results of a screening test performed within two weeks prior to enrollment and remained in that group regardless of subsequent test results. Four of the cats in the FeLV-positive group along with their matched negative controls were removed due to signs of stress during veterinary visits for blood collection, bringing the final totals for each group to 127 cats. The average age of cats at the time of enrollment to the study was 16.5 months for both positive and negative control groups (range 2.1–120 months). Male cats (59%) outnumbered female cats in this population. 

### 3.2. Laboratory Testing

At the time of enrollment, 121/127 cats were positive for p27 antigen by the POC ELISA performed at the laboratory using anticoagulated whole blood (Table 1). Of these, 118/121 (97.5%) POC p27 antigen positive cats were confirmed as FeLV positive using the combination of microtiter plate ELISA for p27 antigen and real-time PCR for proviral DNA. Considering only the microtiter plate ELISA and real-time PCR results, 119/127 cats within the FeLV-positive group were determined to be p27 antigen or proviral DNA positive; 100 cats were positive for both, sixteen cats were only positive for FeLV p27 antigen, and three cats were only positive for FeLV proviral DNA (Table 1). The eight remaining cats in the FeLV-positive group that had originally tested positive for p27 antigen on the POC test performed in the shelter at the time of screening were found to be negative for p27 antigen by microtiter plate ELISA and proviral DNA at the time of enrollment. Five of the eight cats also tested negative on the POC test performed in the laboratory at the time of enrollment, indicating that three positive POC p27 antigen tests could not be confirmed with subsequent testing (possible false positives). The 127 negative control cats all tested negative for p27 antigen by the POC test and microtiter plate ELISA at the time of study enrollment. However, two of the negative control cats (2/127, 1.6%) tested positive for FeLV proviral DNA by PCR (quantitative values of 4.0 × 10^3^ and 2.5 × 10^3^ copies/mL).

### 3.3. Determination of Cutoff Values

All samples with qualitative positive results generated during the six-month study period from either the microtiter plate ELISA for p27 antigen or the real-time PCR for FeLV proviral DNA (*n* = 694) were classified as concordant or discordant. An iterative ROC analysis was performed, as described in the methods, that resulted in a distribution of 500 pairs of p27 antigen and proviral DNA cutoff values from which the point estimates (distribution means) and 95% confidence intervals were calculated. For each partition, the procedure converged within a few iterations. Sensitivity analysis (varying the sample size of selected concordant observations (200–250) and number of random partitions of concordant samples (100–500)) confirmed stability of acquired estimates. The concentration of p27 antigen and the proviral DNA load that best distinguished samples with concordant and discordant results was determined to be 37.5 ng/mL (95% CI 34.5–41.5) and 4.0 × 10^5^ copies/mL (95% CI 6.3 × 10^4^–1.6 × 10^6^), respectively.

**Figure 1:**
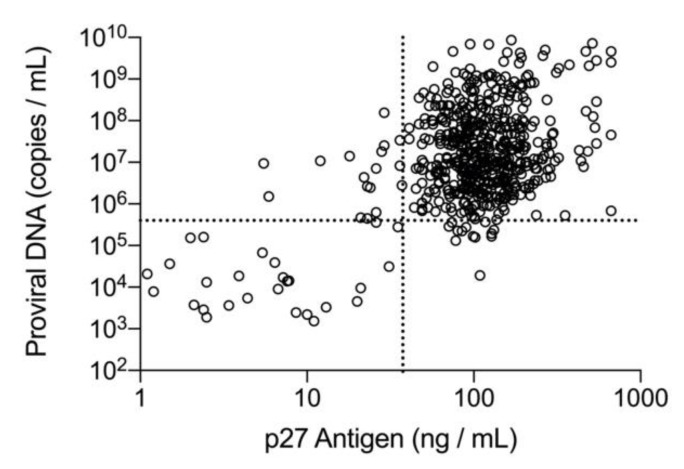
Samples with concordant positive results for p27 antigen by microtiter plate enzyme-linked immunosorbent assay (ELISA) and proviral DNA by real-time polymerase chain reaction (PCR) are plotted relative to the cutoff values established by the receiver operating characteristic (ROC) analysis. Results from 569 samples obtained from enrollment to 6 months are shown. Horizontal dashed line for the proviral DNA is placed at 4.0 × 10^5^ copies/mL. Vertical dashed line for the log of p27 antigen concentration is placed at 37.5 ng/mL.

**Figure 2:**
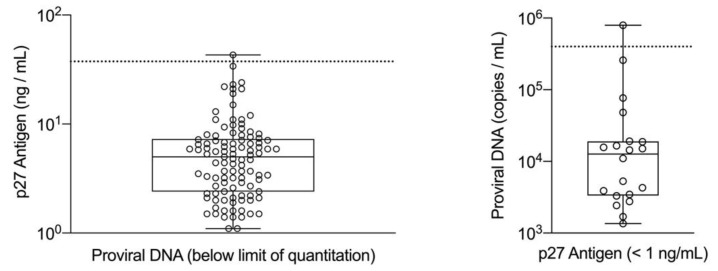
Samples with discordant qualitative test results are graphed separately for p27 antigen positive/proviral DNA negative (**left**, *n* = 105) and for p27 antigen negative/proviral DNA positive (**right**, *n* = 20). Cutoff values from the ROC analysis are represented by horizontal dashed lines at 37.5 ng/mL of p27 antigen (**left**) and 4.0 × 10^5^ copies/mL for proviral DNA (**right**).

For those samples that were positive by both methods (*n* = 569), the results were graphed to evaluate the ROC cutoff values and to assess the association between p27 antigen concentration and number of copies of proviral DNA (Figure 1). The majority of the samples with concordant qualitative results had quantitative values above each of the cutoffs determined by the ROC analysis reflecting consistently high p27 antigen concentrations and high proviral DNA loads in this group of cats (upper right quadrant, *n* = 514). There were 26 samples that despite having concordant positive results for p27 antigen and proviral DNA loads, had quantitative values that were low and fell below the ROC values for both p27 antigen and proviral DNA (lower left quadrant). There were 29 concordant samples that tested above the cutoff on one assay but below the cutoff on the other (11 with high p27 antigen and low proviral loads, and 18 with low p27 antigen and high proviral loads). For the discordant samples (*n* = 125), positive results for p27 antigen concentration and number of copies of proviral DNA are plotted separately relative to the respective cutoff value determined by the ROC analysis (Figure 2). Nearly all of these results fell below their respective cutoff value and within the quantitative range of those concordant samples exhibiting low concentrations of p27 antigen and low copy numbers of proviral DNA. Only two cats, with one sample each, had discordant qualitative results and a quantitative value that was above the ROC cutoff values (one for p27 antigen at 43 ng/mL and one for proviral DNA at 7.9 × 10^5^ copies/mL), making this an uncommon test result in this population.

### 3.4. Longitudinal Results

Finally, the ROC cutoff values were applied to the quantitative results for FeLV-positive cats at enrollment (time 0) to evaluate an association with survival over the four-year study period (Table 2). A total of 87 cats were classified as high positive (both p27 antigen and proviral PCR values above the cutoffs) and 27 cats were classified as low positive (one or both qualitative results for p27 antigen on the microtiter plate ELISA and proviral DNA were positive, but the quantitative values were equal to or below the cutoffs). Eight cats were classified as presumed-negative because both p27 antigen on the microtiter plate ELISA and proviral DNA PCR were negative. There were five cats that had concordant positive results for p27 antigen and proviral DNA, but one quantitative result was above, and one result was below the values determined from the ROC analysis (Table 3). These cats were, then, classified according to the proviral DNA loads, bringing the total number of cats classified as high positive to 90 and as low positive to 29.

**Figure 3:**
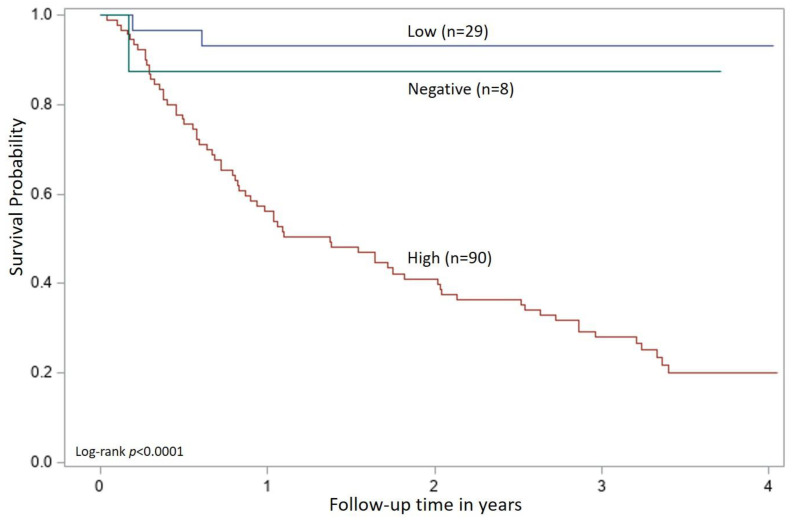
Kaplan–Meier survival curves for cats in the FeLV-positive group and categorized as low positive (Low), high positive (High), or presumed-negative (Negative). Each cat was categorized by applying the cutoff values from the ROC analysis to the test results obtained at the time of enrollment (time 0).

Outcome information was available for all but four cats that were lost to follow-up (one high positive, two low positive, and one presumed-negative) and the last date of contact at which the cat was known to be alive was used for the analysis. In the four-year period during which outcome data was collected, a median survival from the time of enrollment of 1.37 years (95% CI 0.83–2.02) was reached for the high positive group but a median survival was not yet reached for the other two groups (Figure 3). The log-rank test for the Kaplan–Meier analysis reached statistical significance between the groups (*p* < 0.0001). The high positive group had 24.4% survival (68 cats died) at 4 years as compared with 93.1% survival (two cats died) in the low positive group and 87.5% survival (one cat died) in the presumed-negative group. All cats in the presumed-negative group had a positive POC test result for p27 antigen within two weeks of enrollment but tested negative by the microtiter plate ELISA for p27 antigen and by PCR for proviral DNA at enrollment. 

The 29 cats that were classified as low positive based on the cutoffs established by the ROC analysis had variable results over the six-month period of follow-up testing (Table 4). Half (15/29; 51%) of the cats in this group had testing events in which both p27 antigen and proviral DNA results were concordant and qualitatively positive (Table 4, results in bold). The two cats that demonstrated concordant qualitative positive results at each visit (APA030 and APA031), but with classification fluctuating between high positive and low positive, have both died. With the exception of eight monthly testing events that yielded a classification of high positive, the remaining 156 positive monthly testing events from these 29 cats were classified as low positive (156/164, 95%). There were 12/29 cats that only tested positive for p27 antigen, and half of these cats had detectable p27 antigen above the limit of quantitation at every visit. Only one of the 29 cats tested positive for only proviral DNA after an initial positive p27 antigen screening test within two weeks of enrollment to the study. It is also evident from this data that 14 of these infected cats had negative test results at one or more time points even when using the combination of microtiter plate ELISA for p27 antigen and real-time PCR for proviral DNA. Hence, the reason that the eight cats with a positive screening result for p27 antigen within two weeks enrollment but negative results for p27 antigen by microtiter plate ELISA and no detectable proviral DNA in circulation at the time of enrollment were classified as presumed-negative. During the initial six months of the study, none of these cats had proviral DNA detected by PCR. Only one cat had p27 antigen detected by the POC test and microtiter plate ELISA and this cat died before the third month of the study. 

## 4. Discussion

This four-year, prospective study of cats naturally infected with FeLV demonstrated an association between specific cutoff values of p27 antigen concentration and proviral DNA loads and long-term survival. By using the qualitative test results for p27 antigen and proviral DNA, each sample could be assigned to a concordant or discordant category. The categorical groups served as the foundation for the iterative ROC analysis which established quantitative estimates of each measure that could be used to distinguish high positive and low positive cats. The rationale for this approach was based upon the biology of the disease. Cats with progressive infections have been found to have higher levels of p27 antigen, as well as more copies of proviral DNA in circulation and more consistently demonstrate concordant positive results for these measures [5,11,20,25]. Cats with regressive infections, however, have been shown to have both transient detection as well as lower levels of p27 antigen and fewer copies of proviral DNA in circulation which contribute to a higher frequency of discordant test results [5,11,20,25,35]. The current prospective study of naturally infected cats suggests that standardized measures of p27 antigen concentration and proviral DNA loads provide objective evidence to help differentiate progressive and regressive courses of FeLV infections and inform prognosis for long-term survival. 

In this study, cats were tested monthly for six months, and then followed for over three years to assess survival. Using the test results obtained at the time of enrollment (time 0) to characterize a patient’s FeLV-infection course, cats that tested positive for both p27 antigen and proviral DNA and had quantitative results that exceeded the value determined by the ROC analysis were found to have a statistically significant reduction in survival time. High positive cats had a median survival of 1.37 years from the time of enrollment with an upper bound of the 95% confidence interval at 2.0 years. A survival time of three to five years post-infection has been described in the literature for cats with progressive infections and FeLV-associated disease [5,17,36,37,38]. Conversely, cats classified as low positive based on their test results at enrollment had a longer survival and fewer deaths with over 90% still alive at last follow-up. Again, these results are consistent with descriptions of regressive and focal infections in which the immune response to the virus is more robust and limits the extent of the infection [5,18,37]. This study has advanced our understanding of the relationship between p27 antigen concentration, proviral DNA loads, and survival in naturally infected cats, and may provide clinicians with the first concrete tools for generating prognostic information in FeLV-infected cats. In addition, these two tests, when used together, were helpful for confirming positive p27 antigen screening test results at the time of enrollment. In this case, the tests were able to confirm 118/121 positive POC test results (97.5%) that were performed with EDTA-anticoagulated whole blood.

Although concordant and discordant qualitative results were initially used to classify the samples, this alone does not appear to be sufficient for determining the FeLV course or predicting survival. Within the group of cats classified as low positive, 51% (15/29) of the cats had at least one testing event that produced concordant positive qualitative results for p27 antigen and proviral DNA. Because antigenemia has long been associated with viremia, these cats might be assumed to have a progressive clinical course [5,25,39,40]. However, the findings from this study support previous observations that low levels of p27 antigen, as well as low copy numbers of proviral DNA in circulation, may be an indication of an effective immune response [5,10,14]. Cats with positive results for both p27 antigen and proviral DNA should not be assumed to have a progressive infection or a worse prognosis. A transient antigenemia in conjunction with persistent circulating proviral DNA is more commonly associated with regressive (or latent) infections [39,40,41]. However, the low positive cats in this study were more often positive for p27 antigen than proviral DNA. In a concurrent study of these low positive cats, the subset defined as having regressive infections had virus neutralizing antibodies and higher antibody reactivity to the FeLV-SU protein suggesting the presence of active immune control [42]. These results are consistent with previous studies that demonstrated robust antibody responses in regressively infected cats [15,25]. However, antibody responses in cats with focal and abortive infections have been reported to be more variable, complicating the interpretation of a positive or negative antibody result to inform the course of an FeLV infection [9,11,15,16]. It was also observed that several of the cats in this low positive group tested negative for both p27 antigen and proviral DNA at the end of the six-month period. These cats may have established regressive infections with p27 antigen and proviral DNA below the level of detection, as previously described [9,11,18,37]. Further longitudinal follow-up is underway to determine the long-term survival of these cats.

The proportion of cats and test results that were positive for proviral DNA and negative for p27 antigen was lower in this study as compared with previous publications [25,26,43,44,45]. At time of enrollment, only two of the negative control cats and three of the FeLV-positive cats tested positive for only proviral DNA when tested by the laboratory (5/254, 2.0%). With the introduction of a real-time PCR for the exogenous form of FeLV, cats that were previously thought to have cleared an FeLV infection were found to harbor proviral DNA copies of FeLV in the blood [18,20]. As many as 10% of field cats in a Swiss study were suspected of regressive infections because they tested positive for proviral DNA in the absence of detectable p27 antigen [25]. A recent study from Australia found that approximately 5% of cats tested positive for proviral DNA only [43]. The lower proportion of p27 antigen negative/PCR positive cats found within this study may reflect differences in geography, the study population, or screening methods, including assay sensitivity and sample types, used to identify cats eligible for the study. Additionally, a PCR assay less sensitive for samples containing low copy numbers of proviral DNA could contribute to this finding. While most real-time PCR assays should perform similarly on the majority of samples, differences in assay reagents, instrumentation, and cycling conditions could impact the limit of quantitation. Nevertheless, 22/29 cats in the low positive group tested positive for p27 antigen and negative for proviral DNA at least once during the initial six months of the study. These positive results were confirmed to be specific for p27 antigen using FeLV-specific antisera and may reflect the sensitivity of the ELISA used in this study [23,27]. 

Some of the early studies that identified p27 antigen positive cats without evidence of viremia or proviral DNA were performed before the introduction of real-time qPCR assays [14,15,35]. Although today’s more sensitive molecular assays raise questions about these early observations, it is important to recognize that p27 antigen ELISA performance has also benefited from technological improvements such as microtiter plate plastics, plate reader technology, antibody purification and conjugation methods, and sophisticated tools for quality control. The original p27 antigen ELISA was described to have a cutoff of 200 ng/mL as compared with the assay used in the current study which utilized a cutoff of 1 ng/mL [15]. In studies where p27 antigen negative and proviral DNA positive cats were identified, it is possible that cats with low concentrations of p27 antigen were interpreted as negative due to the limit of detection or cutoff value of the ELISA used [5,21,39]. The reduced frequency of p27 antigen negative/PCR positive cats observed in the current study may have been due to the detection of more cats with low concentrations of p27 antigen.

Through a productive collaboration with the shelter, staff, foster homes and adopters, this study benefited from a robust population of samples regularly collected from the same group of cats over time. Blinded testing of these samples generated both categorical and quantitative data that could be used for statistical analysis. The repeated measures ROC analysis was based upon the hypothesis of ergodicity assuming that repeated measurements from a few representative animals taken over the course of a disease are exchangeable and informative as much as a single snapshot taken from a larger population, where individual samples from different animals reveal different stages of disease development [46]. This served as the foundation for the joined analysis of repeated observations (samples) taken from different animals at different times. Being able to leverage all the quantitative data while meeting the requirements of independence dictated by the traditional ROC analysis generated data driven point estimates and narrower confidence intervals for interpretation. The estimated optimal ROC cutoff values assume false positives (attributing high status to cats with active immune control) and false negatives (attributing low status to cats with a failure of immune control) to be equally detrimental. However, from a medical perspective, it may be advantageous to reduce false positive (at the expense of increasing false negative) rates by assigning lower weights to false positive events for the purpose of the ROC analysis. This would help to minimize an error of calling some cats with low p27 antigen/proviral loads as falsely high but would potentially call some cats with high p27 antigen/proviral loads as low. While further clinical experience and additional patient outcome data will help to refine these values, the ROC cutoff values for p27 antigen concentration and proviral DNA loads determined in the current study were similar to those generated from a convenience population of feline whole blood samples submitted to a commercial reference laboratory for real-time PCR testing [27].

Studies of cats with naturally acquired FeLV infections are valuable for providing practical medical guidance and expectations of outcome. These cats represent an outbred population infected with field strains of FeLV via natural exposure routes. As pets, they live under real-world conditions and in a variety of household environments. Thus, they reflect the experience that cat owners can expect for their own cats. However, there were some limitations associated with naturally infected shelter cats, including lack of knowledge of how long they had been infected prior to entering the shelter. In addition, the inability to follow the negative control cohort over time could have contributed to some verification bias because it is not known how many of the negative control cats might have had early infections and would have had changes to their results if retested monthly for 6 months. To minimize the bias, all cats in the negative control group had all the same tests performed as the positive cats at the time of enrollment. Experimental infections have demonstrated that it may take up to 8 weeks post-infection before differences in p27 antigen levels and proviral DNA loads are apparent between progressive and regressive cats [39]. If a cat is tested very early post-infection, retesting would be essential to establish an accurate clinical course of infection based upon the quantitative cutoff values established in this study. While it is often difficult to obtain owner compliance with repeated testing of healthy cats, the benefit of follow-up testing is a more precise knowledge of a cat’s status and likely clinical course. Outcome information from the FeLV-positive group was limited to the question of survival in order to gain optimal compliance. Cause of death was not confirmed for most of these cats, and just because a cat was deceased upon follow-up did not necessarily mean that it died of an FeLV-related cause. An additional limitation of the study is that there is no “gold standard” for the diagnosis of FeLV that is consistently reliable throughout all courses of infection. Unfortunately, even virus isolation and PCR for viral RNA, which perform well during times of viremia, are less likely to detect cats with regressive or focal infections [5,37]. 

## 5. Conclusions

Using the quantitative results for p27 antigen concentration and proviral DNA loads, this study was able to establish cutoff values to classify cats into high positive and low positive groups that were associated with significantly different survival times over the four-year study period. Even though the course of an FeLV infection is very much dependent on the immune status of the cat, the vast majority of cats confirmed to be infected at time 0 remained infected throughout the 6-month testing period and tended to maintain their high positive (higher risk for early death) or low positive (lower risk for early death) status. Quantitative values for p27 antigen concentration and proviral DNA loads provide an objective means to confirm POC screening results and to help monitor FeLV-infected patients over time. These results provide new support for practical implementation of international guidelines for optimizing management of both infected and uninfected cats, including accurate diagnosis, veterinary monitoring, and segregating infected cats into single-cat households or with other FeLV-infected cats.

## Figures and Tables

**Table 1:** Feline leukemia virus (FeLV) p27 antigen (Ag) and proviral DNA results for the enrolled population. Screening with the point-of-care (POC) test at the shelter occurred within the two weeks prior to study enrollment. The POC test was repeated at the laboratory with the sample obtained at the time of enrollment (time 0).

Group	Screening at the Shelter		Laboratory ^‡^ Testing at the Time of Study Enrollment					
	POC Test Result for p27 Ag		POC Test Result for p27 Ag		Microtiter Plate ELISA Result for p27 Ag and PCR Result for Proviral DNA			
	Pos	Neg	Pos	Neg	Ag Pos/PCR Pos	Ag Pos/PCR Neg	Ag Neg/PCR Pos	Ag Neg/PCR Neg
FeLV-positive	127	0	121	6	100	16	3 *	8 **
FeLV-negative	0	127	0	127	0	0	2	125

**^‡^** Laboratory testing was performed at IDEXX R&D (Ag) and IDEXX Reference Laboratories (PCR). * One of the three cats tested negative for p27 antigen by the POC test using EDTA-anticoagulated whole blood in the laboratory. ** Five of the eight cats tested negative for p27 antigen by the POC test using EDTA-anticoagulated whole blood in the laboratory.

**Table 2:** Classification of 119 cats in the FeLV-positive group at the time of enrollment using the cutoff values determined from the ROC analysis. When the qualitative results for a sample were positive for both assays but quantitative values were not both above or not both below the respective cutoffs, the quantitative result for proviral DNA (boldface) was used for classification.

Classification	*n*	Qualitative Results		Quantitative Results	
		Microtiter Plate ELISA for p27 Ag	Real-Time PCR for Proviral DNA	p27 Ag (ng/mL)	Proviral DNA (Copies/mL)
High positive (likely progressive infection)	87	Positive	Positive	>37.5	>4.0 × 10^5^
	3	Positive	Positive	≤37.5	**>4.0 × 10^5^**
Low positive (likely regressive infection)	8	Positive	Positive	≤37.5	≤4.0 × 10^5^
	2	Positive	Positive	>37.5	**≤4.0 × 10^5^**
	16	Positive	Negative	≤37.5	NA
	3	Negative	Positive	NA	≤4.0 × 10^5^

**Table 3:** At enrollment, five cats with positive qualitative results for both p27 antigen and proviral DNA had quantitative results that were not both above and not both below the determined ROC cutoff values of 37.5 ng/mL of p27 antigen and 4.0 × 10^5^ copies/mL of proviral DNA. Classification as high positive or low positive was assigned based upon the quantitative value for copy numbers of proviral DNA.

Cat ID	Qualitative Results		Quantitative Results		Classification	Outcome (Years)
	Microtiter Plate ELISA for p27 Ag	Real-Time PCR for Proviral DNA	p27 Ag (ng/mL)	Proviral DNA (Copies/mL)		
APA005	Positive	Positive	29	1.6 × 10^8^	High	Dead (0.2)
APA030	Positive	Positive	90	2.5 × 10^5^	Low	Dead (0.2)
APA031	Positive	Positive	78	1.3 × 10^5^	Low	Dead (0.6)
APA061	Positive	Positive	26	6.3 × 10^6^	High	Alive (3.6)
APA088	Positive	Positive	22	4.0 × 10^6^	High	Alive (3.3)

**Table 4:** Longitudinal test results for the 29 FeLV-positive cats classified as low positive based upon the ROC cutoff values applied to the test results obtained in the laboratory at the time of enrollment. Time points with concordant, qualitative positive results between p27 antigen and proviral DNA are shown in bold. Grey boxes indicate missing samples. (+, positive; −, negative; L, low positive; H, high positive; †, dead).

	p27 Antigen								Proviral DNA						
	POC	Microtiter Plate ELISA							Real-Time PCR						
Time (months)	0	0	1	2	3	4	5	6	0	1	2	3	4	5	6
APA030 †	+	**H**	**H**						**L**	**H**					
APA031 †	+	**H**	**H**	**H**	**H**	**H**	**H**	**H**	**L**	**H**	**L**	**L**	**H**	**L**	**H**
APA016	−	−	**L**	−	−	−	−	−	L	**H**	L	L	L	−	−
APA020	+	−	**L**	**L**	−	−	−	−	L	**L**	**L**	L	L	L	L
APA027	+	**L**	**L**	L	**L**	−	−	−	**L**	**L**	−	**L**	−	−	L
APA042	+	L	L	L	**L**	L	L	L	−	−	−	**L**	−	−	−
APA047	+	L	L	L	L	L	**L**	L	−	−	−	−	−	**L**	−
APA052	+	**L**	**L**	L	L	L	**L**	L	**L**	**L**	−	−	−	**L**	−
APA100	+	**L**	L	L	L	L	−	−	**L**	−	−	−	−	−	−
APA104	+	**L**	L	L	L	L	L	L	**L**	−	−	−	−	−	−
APA111	+	L	**L**	L	L	**L**	−	−	−	**L**	−	−	**L**	L	L
APA121	+	**L**	L	L	**L**	**L**	L	−	**L**	−	−	**L**	**L**	−	−
APA122	+	**L**	**L**	L	L	**L**	**L**	L	**L**	**L**	−	−	**L**	**L**	−
APA124	+	L	L	L	L	−	−	L	−	−	−	−	L	−	−
APA127	+	**L**	**L**	**L**	**L**	−	−	L	**L**	**L**	**H**	**L**	−	L	L
APA134	+	**L**	**L**	−	−	−	−	−	**L**	**H**	H	L	−	−	−
APA004	+	L	L	L	L	L	L	L	−	−	−	−	−	−	−
APA007	+	L	−	−	−	−	−	−	−	−	−	−	−	−	−
APA043	+	L	L	L	L	L	L	L	−	−	−	−	−	−	−
APA084	+	L	L	L	L	L	L	L	−	−	−	−	−	−	−
APA087	+	L	L	L	L	L	L	−	−	−	−	−	−	−	−
APA092	+	L	L	L	L	L	L	L	−	−	−	−	−	−	−
APA099	+	L	L	L	L	−	L	−	−	−	−	−	−	−	−
APA101	+	L	L	L	−	−	−	−	−	−	−	−	−	−	−
APA103	+	L	L	L	L	−	L	L	−	−	−	−	−	−	−
APA117	+	L	L	L	L	L	L	L	−	−	−	−	−	−	−
APA128	+	L	L	L	L	L	L	L	−	−	−	−		−	−
APA129	+	L	L	−	L	L	−	−	−	−	−	−		−	−
APA059	+	−	−	−	−	−	−	−	L	−	−	−	L	−	−

## Data Availability

Data presented in this study are available upon request from the corresponding author. Data are not publicly available due to the proprietary nature of the commercial assays and privacy associated with ongoing patient monitoring to determine long-term outcome.

## Supplementary Materials

The following are available online at https://www.mdpi.com/1999-4915/13/2/302/s1.
