# Supplementary material for: Feline Leukemia Virus p27 Antigen Concentration and Proviral DNA Load Are Associated with Survival in Naturally Infected Cats
Source: Viruses. 2021 Feb 15;13(2):302. doi: 10.3390/v13020302 (PMC7919025; doi:10.3390/v13020302)
Supplement: Supplementary file 1 [file viruses-13-00302-s001.pdf]

A receiver operating characteristic (ROC) analysis, adjusted to account for correlation between repeated measurements (observations) from the same cat performed over the course of study, was used to establish the point estimates and 95% confidence intervals for the p27 antigen concentration and the FeLV proviral DNA loads that provided the optimal discrimination (based on Youden criteria) between samples with qualitative concordant (both positive) and qualitative discordant (one positive and one negative) results [1-2]. All samples with positive qualitative results for p27 antigen by microtiter plate ELISA or proviral DNA by real-time PCR were included in the analysis (n=694). Samples were classified as concordant when the p27 antigen and proviral DNA results were both positive. Discordant samples were classified as either p27 antigen positive/PCR negative or p27 antigen negative/PCR positive. To meet the requirement of independence, two non-overlapping sets of 250 observations were randomly selected (using simple random sampling) from the concordant data. The Floyd's ordered hash table algorithm was used for simple random sampling [3]. Random number generation was performed using Mersenne twister uniform random number generator [4]. Within each combined sample set, two smaller subsets of equal size of independent observations suitable for ROC analysis were generated, with the first observation on each selected cat being included into the first subset and the last observation on the same cat being included into the second subset. If only one observation was present for a selected cat, it was included both into the first and into the second subsets. An overlap between the two subsets was found to be about 20% (95% CI: 15%-25%) for 'concordant + Ag positive/PCR negative discordant' data and about 22% (95% CI: 16%-29%) for 'concordant + p27 antigen negative/PCR positive discordant' data.

Using the qualitative concordant/discordant sample classification, a first iteration p27 antigen concentration ROC analysis was performed on the **first** subset of 'concordant + Ag positive/PCR negative discordant' data, Youden cutoff calculated, and samples in the **second** subset of 'concordant + Ag positive/PCR negative discordant' data reclassified based on the calculated cutoff. These newly classified samples were then used in the second iteration ROC analysis on the **second** subset to produce a new cutoff that was used to reclassify the samples in the **first** subset of data. This procedure continued until convergence criterium (≤0.0001) for relative difference (defined as absolute difference divided by the mean) in consecutively calculated cutoffs was satisfied. If the 'odd' (first, third, etc.) iteration cutoff resulted in a full data separation (ROC AUC=1) of the second subset, or if the 'even' (second, fourth, etc.) iteration cutoff resulted in a full data separation of the first subset, the iteration process stopped, and that cutoff was chosen.

Our choice of convergence criteria was based on a realistic precision we would like to achieve on antigen and proviral DNA concentration cutoffs. We measured Ag concentration with precision of ±0.1 ng/mL, which corresponds to a relative difference (absolute difference divided by the mean value) of about 0.1/37= 0.003 around the cutoff. For log of proviral DNA copies/ul, we were looking for ±0.1 precision, which corresponds to about 0.1/5.6=0.02 relative difference around the cutoff. Consequently, the common

convergence criteria for relative difference in consecutive cutoff iterations should be satisfied on a scale of ≤0.001.

After the p27 antigen cutoff was determined, the first subset of 'concordant + p27 antigen negative/PCR positive discordant' data was classified using both that p27 antigen cutoff and the qualitative concordant/discordant sample status. A sample was classified as 'discordant' either when p27 antigen concentration fell below the p27 antigen cutoff or when it was called discordant by the qualitative test. Then, iterations of the proviral DNA concentration ROC analysis using the first and the second subsets of 'concordant + p27 antigen negative/PCR positive discordant' data continued as described above until either convergence or full data separation, whichever comes first. Using proviral DNA logs of copies/mL improved normality of distribution and allowed for a better precision in calculating the cutoff values.

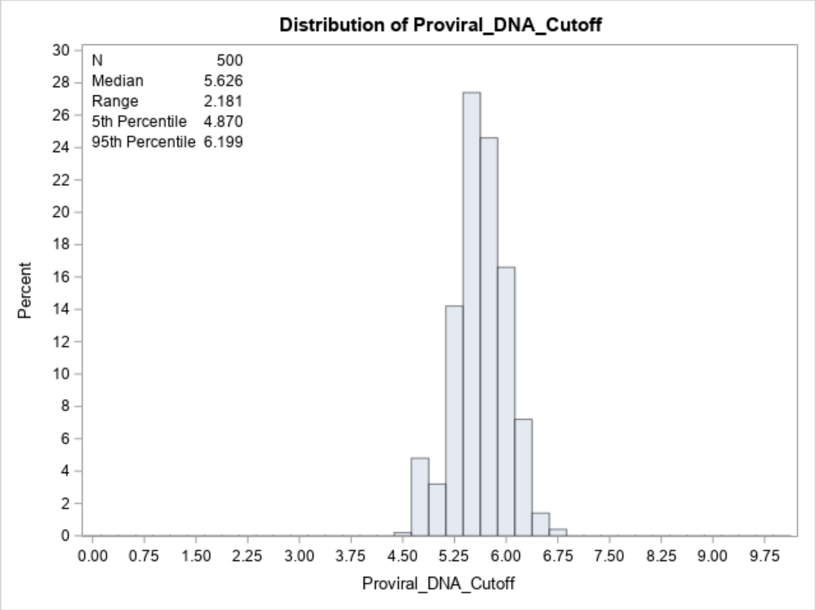

Thus, each random partition of the concordant data was to produce a single pair of p27 antigen and proviral DNA cutoffs. The described analysis was repeated 500 times, each time with a new partition of 250 + 250 pair of concordant sample sets, to produce a distribution of 500 pairs of p27 antigen and proviral DNA cutoff values. The distributions of calculated cutoffs were used to determine the point estimates (distribution median) and 95% confidence intervals of the cutoff values for the p27 antigen concentration and proviral DNA load.

1. Kamarudin, A.N.; Cox, T.; Kolamunnage-Dona, R. Time-dependent ROC curve analysis in medical research: Current methods and applications. *BMC Med. Res. Methodol.* 2017, *17*, 53.

2. Lasko, T.A.; Bhagwat, J.G.; Zou, K.H.; Ohno-Machado, L. The use of receiver operating characteristic curves in biomedical informatics. *J. Biomed. Inform.* 2005, *38*, 404–415

3. Bentley, J. L., and Floyd, R. W. (1987). Programming Pearls: A Sample of Brilliance. *Communications of the Association for Computing Machinery* 30:754–757

4. Matsumoto, M., and Nishimura, T. (1998). Mersenne Twister: A 623-Dimensionally Equidistributed Uniform Pseudo-random Number Generator. *ACM Transactions on Modeling and Computer Simulation* 8:3–30.
